# CircRNA circTIAM1 promotes papillary thyroid cancer progression through the miR-646/HNRNPA1 signaling pathway

**DOI:** 10.1038/s41420-021-00798-1

**Published:** 2022-01-12

**Authors:** Deguang Zhang, Li Tao, Nizheng Xu, Xiaoxiao Lu, Jianle Wang, Gaofei He, Qinghu Tang, Kangmao Huang, Shuying Shen, Junjie Chu

**Affiliations:** 1 Department of head and neck surgery, Institute of Micro-Invasive Surgery of Zhejiang University, Sir Run Run Shaw Hospital, Medical School, Zhejiang University, Hangzhou, People’s Republic of China; 2 Department of orthopaedic surgery, Sir Run Run Shaw Hospital, Zhejiang University school of Medicine & Key laboratory of Musculoskeletal system Degeneration and regeneration Translational research of Zhejiang Province, 3 east Qingchun road, Hangzhou, 310016 People’s Republic of China; 3 Department of general surgery, People’s Hospital of Linghu, Nanxun District, Huzhou, Zhejiang Province People’s Republic of China

**Keywords:** Thyroid cancer, Tumour angiogenesis

## Abstract

Papillary thyroid cancer (PTC) is a common endocrine tumor with a rapidly increasing incidence in recent years. Although the majority of PTCs are relatively indolent and have a good prognosis, a certain proportion is highly aggressive with lymphatic metastasis, iodine resistance, and easy recurrence. Circular RNAs (circRNAs) are a class of noncoding RNAs that are linked to a variety of tumor processes in several cancers, including PTC. In the current study, circRNA high-throughput sequencing was performed to identify alterations in circRNA expression levels in PTC tissues. circTIAM1 was then selected because of its increased expression in PTC and association with apoptosis, proliferation, and migration of PTC cells in vitro and in vivo. Mechanistically, circTIAM1 acted as a sponge of microRNA-646 and functioned in PTC by targeting miR-646 and heterogeneous ribonucleoprotein A1. Fluorescence in situ hybridization and dual-luciferase reporter assays further confirmed these connections. Overall, our results reveal an important oncogenic role of circTIAM1 in PTC and may represent a potentially therapeutic target against PTC progression.

## Introduction

Thyroid cancer is the most prevalent malignant tumor of the endocrine system and has received increasing attention from researchers owing to its rapidly increasing incidence [1]. In the past 30 years, the incidence of thyroid cancer has nearly tripled, and the annual rate of increase in new cases has stabilized at approximately 5% [2]. Papillary thyroid carcinoma (PTC) accounts for almost 85% of these cases [3]. Although most patients with PTC have a good prognosis, those who have lymph node metastasis, distant metastasis, extracapsular invasion, or other high-risk factors still have a high recurrence rate after surgical treatment, which seriously reduces the quality of patients’ lives and even leads to death [4, 5]. Therefore, it is urgent to understand the underlying mechanism of the progression of PTC and provide more perspectives for exploring novel therapies for thyroid cancer.

A part of RNAs transcribed through the human genome are noncoding RNAs, including microRNAs (miRNAs), circular RNAs (circRNAs), and long noncoding RNAs [6–8]. CircRNAs lack the 5ʹ cap and 3ʹ polyA tail, forming a covalently closed-loop structure, which allows them to maintain relatively high stability [9]. CircRNAs are widely involved in the pathophysiological processes, mainly by sponging miRNAs or interacting with different RNA-binding proteins (RBPs) [10–12]. Numerous studies have revealed the inseparable relationship between circRNAs and various malignant tumors, including glioma, gastric cancer, osteosarcoma, lung cancer, and hepatocellular carcinoma [7, 13–16]. In addition, several circRNAs were found to be enriched in PTC tissues and affect PTC progression, which highlights the importance of circRNAs in thyroid cancer.

In this study, a novel circRNA, hsa_circ_0061406 (hsa_circTIAM1_026), was found to be differentially expressed between PTC tissues and adjacent paracancerous tissues via high-throughput sequencing. Hsa_circ_0061406, the transcription product of the cell lymphoma invasion and metastasis-inducing factor 1 (TIAM1) gene, is highly expressed in PTC tissues and regulates the level of miRNA-646 (miR-646) by sponging heterogeneous ribonucleoprotein A1 (HNRNPA1) to mediate the proliferation, apoptosis, and migration of PTC cells.

## Results

### Expression profiling of circRNAs in human PTC tissues

**Fig. 1:**
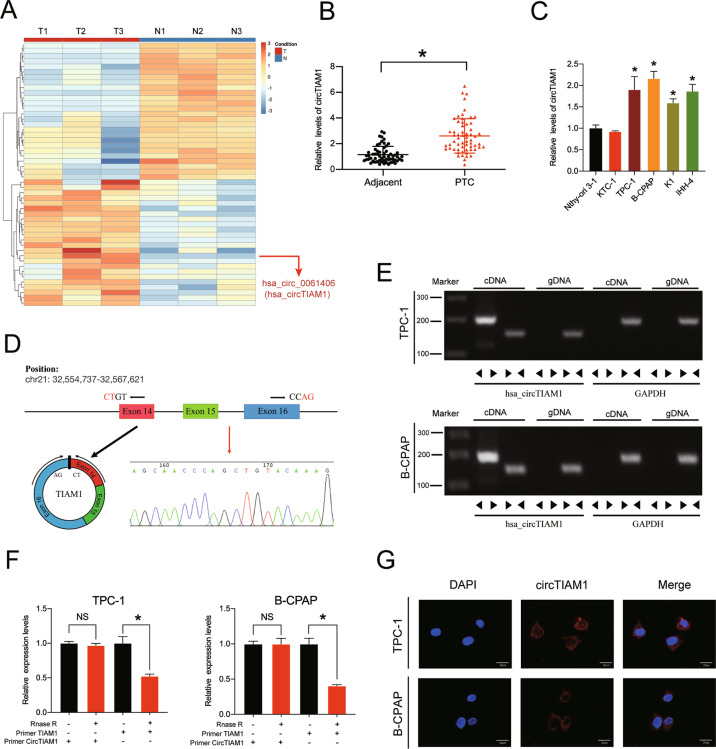
CircTIAM1 validation and expression in PTC tissues and cells. **A** Heatmap of all differentially expressed circRNAs between PTC and paracarcinoma tissues, based on circRNA microarray (TOP 50). **B** CircTIAM1 expression was higher in PTC tissues than para-cancinoma tissues (*n* = 60). *p < 0.05. **C** High levels of circTIAM1 were detected in multiple PTC cell lines compared with Nthy-ori 3-1 cell line. **D** Schematic illustration showing the formation of circTIAM1 via the circularization of TIAM1 exons 14–16 (black arrow). Sanger sequencing demonstrated the the effectiveness of primers and the cyclization characteristics of circTIAM1, the red arrow indicates the splicing sites from head to tail. **E** The presence of circTIAM1 was validated in PTC cells by RT-PCR and represented via agarose gel electrophoresis. Divergent primers amplified circTIAM1 from cDNA, but not from genomic DNA in PTC cells. GAPDH severed as a control. **F** circTIAM1 and TIAM1 expression at mRNA level was analyzed by RT-qPCR after RNase R treatment compared with controls. **G** RNA FISH revealed that circTIAM1 was localized in the cytoplasm of PTC cells. CircTIAM1 probes were labeled with Cy3, and the nuclei were stained with DAPI. Scale bar = 20 μm. Data represent the mean ± SD. Three independent assays were performed (**C** and **F**) (*p < 0.05; Student’s *t*-test).

To identify the circRNAs that play important roles in thyroid cancer, a high-throughput circRNA microarray analysis was performed using three pairs of carcinoma and paracarcinoma tissues from PTC patients (GSE168449). The top 50 most differentially expressed circRNAs were identified by fold-change filtering and Student’s *t*-test. The results are shown as a heatmap and volcano plot (Fig. 1A and Fig. S1A). A detailed list of the top 50 differentially expressed circRNAs is provided in Fig. S1B and hsa_circ_0061406 releaved highest fold-change in the PTC tissues than in the control tissues (log2FoldChange = 7.48) which indicates that it may play a key role in PTC and is worthy of further research. This circRNA is derived from TIAM1 gene and has not been reported to date to the best of our knowledge.

### CircTIAM1 is upregulated in human PTC samples and cell lines

To further verify the contribution of circTIAM1 to the tumorigenesis of PTC, real-time quantitative polymerase chain reaction (RT-qPCR) analysis was performed using RNA obtained from 60 paired PTC tissues and adjacent paracancerous tissues (Fig. 1B). The evaluation of the clinicopathological significance of circTIAM1 revealed that a high level of circTIAM1 expression was associated with unfavorable prognosis (higher tumor-necrosis-metastasis stage, higher proportion of lymph node metastases, and larger tumor sizes), indicating that circTIAM1 might be a novel prognostic biomarker for PTC (Table S1). TPC-1 and B-CPAP PTC cells had the highest enrichment of circTIAM1 compared with that in normal thyroid cells (e.g., Nthy-ori 3-1) and were therefore selected for subsequent investigation (Fig. 1C). Sanger sequencing was performed to confirm the predicted head-to-tail splicing PCR product of circTIAM1 using divergent primers, and the product met the expected length when we designed the primers (Fig. 1D). In addition, convergent and divergent primers were designed to amplify circTIAM1, and circTIAM1 was detected only in cDNA samples, while TIAM1 was detected in both cDNA and genomic DNA samples (Fig. 1E). RNase R was used to treat TIAM1 and circTIAM1 mRNAs in TPC-1 and B-CPAP cells, which showed that circTIAM1 was resistant to RNase R, while the TIAM1 mRNA levels were greatly decreased after RNase R treatment (Fig. 1F). Furthermore, circTIAM1 was abundant in the cytoplasm, as determined by fluorescence in situ hybridization (FISH) assays (Fig. 1G).

### CircTIAM1 regulates the migration, proliferation, and apoptosis of PTC cells

**Fig. 2:**
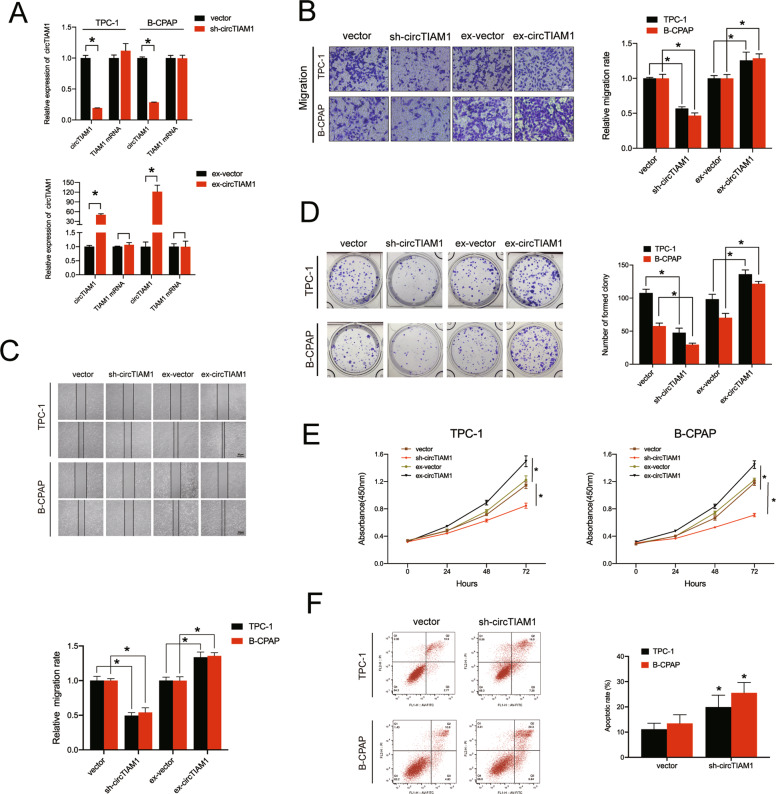
CircTIAM1 affects the migration, proliferation, and apoptosis of PTC cells. **A** TPC-1 and B-CPAP cells were transfected with circTIAM1 short hairpin RNAs or overexpression plasmid, and the expression levels of circTIAM1 and TIAM1 mRNA were measured by RT-qPCR and normalized to GADPH level. **B** The effect of circTIAM1 downregulation or overexpression on cell migration was evaluated by the Transwell migration assay in PTC cells. Scale bar = 100 μm. **C** The wound-healing assay was conducted to evaluate the migration abilities of TPC cells transfected with sh-circTIAM1/ex-circTIAM1 plasmid or vector. **D** Representative images of PTC cells transfected with sh-circTIAM1 or ex-circTIAM1 in colony formation assay. **E** Cell proliferation rate was significantly suppressed in PTC cells with circTIAM1 knockdown, while it was increased with circTIAM1 overexpression. **F** PTC cells were transfected with sh-circTIAM1, and Annexin V-FITC/PI staining was conducted to show the percentage of apoptotic cells. Data represent the mean ± SD. Three independent assays were performed (**A**–**F**) (*p < 0.05; Student’s *t*-test).

To explore the involvement of circTIAM1 in PTC cell functions, circTIAM1 small hairpin RNAs (shRNAs) and circTIAM1 overexpression plasmids were designed and transfected into PTC cells to establish stable circTIAM1-knockdown or -overexpressing cell lines. The transfection efficiency of circTIAM1 and subsequent TIAM1 expression levels were examined by qRT-PCR, and the results showed that knockdown or overexpression of circTIAM1 expression did not influence the TIAM1 mRNA levels (Fig. 2A). The migration abilities of PTC cell lines were sharply decreased by circTIAM1 knockdown as revealed by the Transwell migration and wound healing assays, while high levels of circTIAM1 exhibited the opposite effect (Fig. 2B, C). In addition, colony formation and cell counting kit-8 (CCK-8) assays were conducted to evaluate the proliferative capability of PTC cells. Cells transfected with circTIAM1 shRNA showed a significant reduction in cell proliferation, while compared to non-transfected control cells, circTIAM1 overexpression significantly increased the number of cell clones formed and the cell proliferation rate (Fig. 2D, E). Moreover, the apoptosis assay indicated that circTIAM1 silencing might lead to apoptosis in TPC-1 and B-CPAP cells (Fig. 2F). The collective findings indicate that circTIAM1 promotes the migration, proliferation, and apoptosis of PTC cells in vitro.

### CircTIAM1 serves as a sponge for miR-646 in vitro

**Fig. 3:**
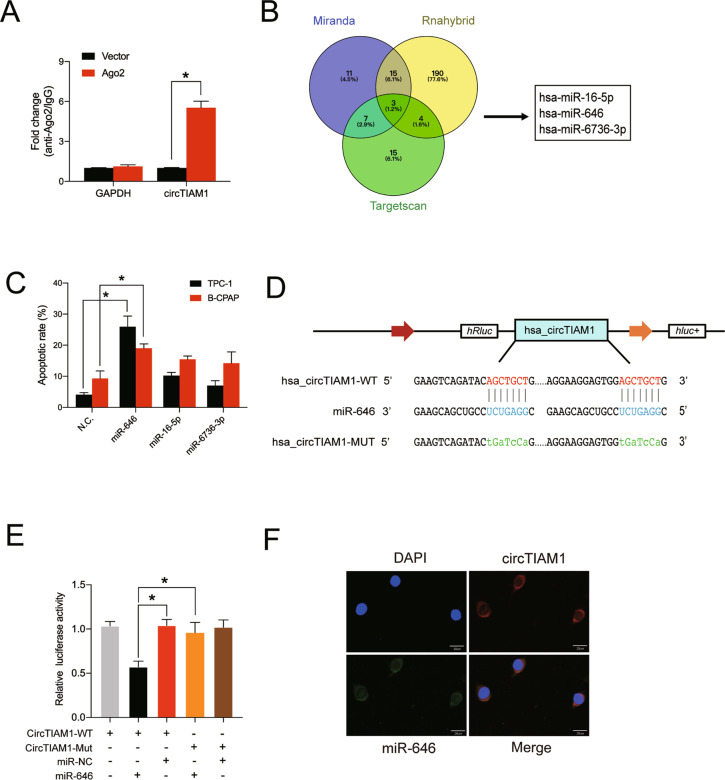
CircTIAM1 serves as a sponge for miR-646 in PTC cells. **A** Ago2 RIP assay was conducted to evaluate circTIAM1 pulled down by the anti-Ago2 antibody in B-CPAP cells transfected with Ago2 plasmid or vector. **B** Schematic diagram showing the potential microRNAs that bind to circTIAM1, revealed by the overlapping of the TargetScan, miRanda, and RNAhybrid databases. **C** TPC-1 and B-CPAP cells were transfected with target microRNA mimics or mimic NC, and the cells were collected and stained with Annexin V-FITC/PI after 48 h. The apoptotic rates were shown as mean ± S.D. **D** Schematic diagram of the binding sequence of circTIAM1 and miR-646. Mutated nucleotides of circTIAM1 are represented in lowercase letters. **E** Luciferase reporter assay was conducted to evaluate the luciferase activity of HEK-293T co-transfected with miR-646 mimics or NC and the luciferase reporter containing wild-type or mutated circTIAM1. **F** FISH revealed co-localization of circTIAM1 and miR-646 in TPC-1 cells. CircTIAM1 probes were labeled with Cy3, miR-646 probes were labeled with FAM, and DAPI was chosen to stain the nucleus (Scale bar = 20 μm). Data represent the mean ± SD. Three independent assays were performed (**A**&**C**) (*p < 0.05; Student’s *t*-test).

Previous research has confirmed that circRNAs can act as miRNA sponges to exert their biological functions in tumors [17–19]. Given that circTIAM1 is enriched and stable in the cytoplasm of PTC cells, it may act as an miRNA sponge to participate in the tumor progression of PTC. To further validate this hypothesis, RNA immunoprecipitation was performed using an antibody against argonaute 2 (Ago2) in B-CPAP cells transfected with Ago2 plasmid or vector. Specific high levels of enrichment of endogenous circTIAM1 were observed in the AGO2 overexpression group compared with the control group (Fig. 3A). Then, we used the TargetScan, RNAhybrid, and miRanda databases to predict the potential miRNA recognition elements in the circTIAM1 sequence, and three miRNAs were identified. An apoptosis assay was conducted, and at 48 h, the overexpression of miR-646 was found to have the most significant impact on cell apoptosis in both TPC-1 and B-CPAP cells and miR-646 was therefore selected for further analysis (Fig. 3C and Fig. S2A). Since circTIAM1 contains two potential target sites for miR-646 (Fig. 3D), luciferase reporters containing circTIAM1 with the sequences of these two mutated potential target sites were established. miR-646 mimics significantly reduced the luciferase reporter activities in the circTIAM1 NC group and thus confirmed their interactions (Fig. 3E). Furthermore, RNA FISH assay showed the co-localization of circTIAM1 and miR-646 in the cytoplasm of TPC-1 cells (Fig. 3F). Collectively, these results indicate that miR-646 is a target of circTIAM1.

### Knockdown of miR-646 reverses the antitumor effects of sh-circTIAM1 on PTC cells

**Fig. 4:**
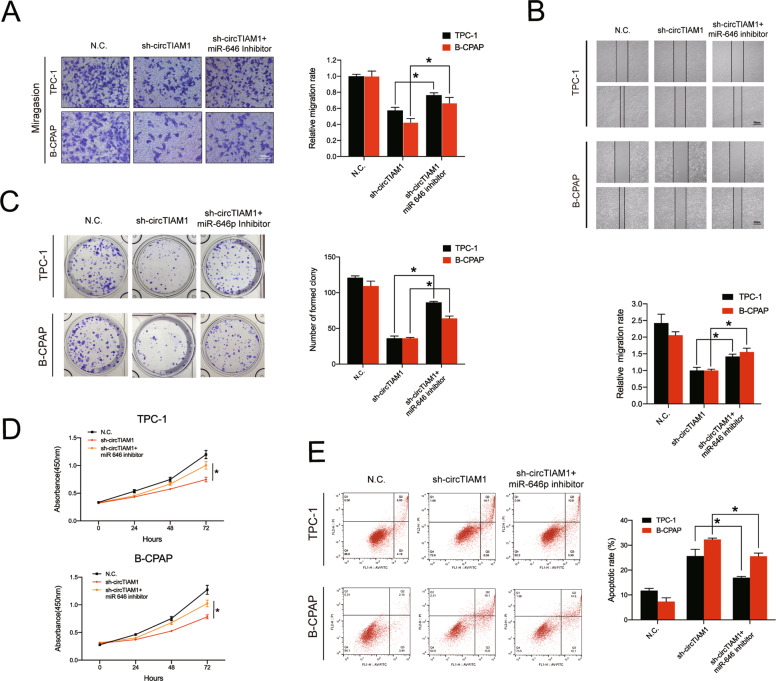
Knockdown of miR-646 reverses the sh-circTIAM1-induced antitumor effects in PTC cells. **A** TPC-1 and B-CPAP cells were transfected with sh-NC or sh-circTIAM1 or co-transfected with sh-circTIAM1 and miR-646 inhibitor, and cell migration was evaluated by Transwell migration assay. Scale bars = 100 μm. **B** The wound-healing assay revealed the reversion of the migration capacity due to low levels of miR-646. **C** miR-646 downregulation stimulated the proliferation capacity of stable circTIAM1-knockdown PTC cells by colony formation assay. **D** The CCK-8 assay exhibited that miR-646 reversed the sh-circTIAM1-induced effects on proliferation capacity. **E** The apoptosis assay was conducted to evaluate the rescue effect of circTIAM1 silencing and miR-646 downregulation on circTIAM1 silencing. Data represent the mean ± SD. Three independent assays were performed (**A**–**E**) (*p < 0.05; Student’s *t*-test).

To confirm the hypothesis that circTIAM1 promotes the tumor progression of PTC mainly by interacting with miR-646, rescue experiments were conducted using TPC-1 and B-CPAP cells that were co-transfected with sh-circTIAM1 or sh-NC and miR-646 inhibitor or control vector. The transfection efficiencies of miR-646 mimics or inhibitor were detected by qRT-PCR (Fig. S2B). The Transwell migration assay indicated that decreased levels of miR-646 expression could reverse the deficiency of cell migration caused by sh-circTIAM1 (Fig. 4A). A similar rescue effect was observed in the wound-healing assay (Fig. 4B). Furthermore, knockdown of miR-646 partly rescued the inhibition of the proliferative ability of sh-circTIAM1 PTC cells, as demonstrated by colony formation assay and CCK-8 assay (Fig. 4C, D). The apoptosis assay indicated that apoptosis deficiency in sh-circTIAM1 TPC-1 and B-CPAP cells could be triggered by miR-646 silencing (Fig. 4E).

### HNRNPA1 is a direct target of miR-646 and an oncogene in PTC

**Fig. 5:**
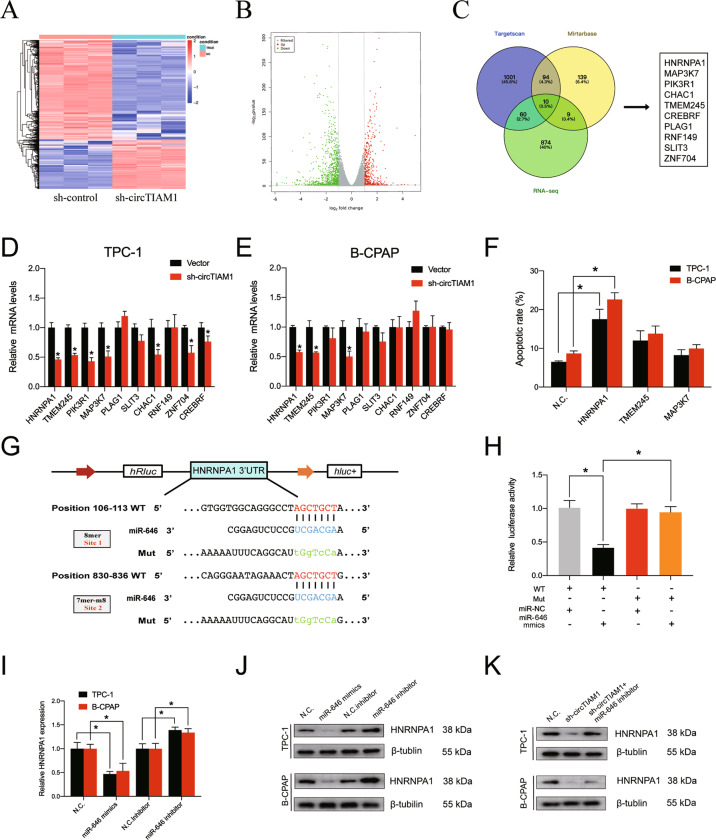
HNRNPA1 serves as a direct target of miR-646. **A** Stably transfected sh-circTIAM1 TPC-1 cells were collected, and high-throughput sequencing was conducted to identify differentially expressed mRNAs. The results are represented as a clustered heatmap. **B** Volcano plots showing significantly overexpressed genes as red dots and significantly downregulated genes as green dots. **C** The overlapping of downregulated mRNAs in sh-circTIAM1-silenced cells and target genes of miR-646 identified from the TargetScan and Mirtabase databases was represented as a flowchart. **D**, **E** qRT-PCR assay was performed to evaluate the expression of 10 selected genes in PTC cells with circTIAM1 silencing or control. **F** The apoptotic rates were detected with PTC cells transfected with HNRNPA1, TMEM245, and MAP3K7 siRNAs. **G** The putative binding sequence of HNRNPA1 3ʹ-UTR and miR-646 was shown, and the mutated binding sequences are represented blow. **H** HEK-293T cells were co-transfected with luciferase reporter containing wild-type or mutated HNRNPA1 and miR-646 mimics or NC. The luciferase activity was then detected, followed by luciferase reporter assay. **I***,* **J** TPC-1, and B-CPAP cells were transfected with miR-646 mimics or inhibitor, and the relative expression of HNRNPA1 was observed in both mRNA level and protein level by qRT-PCR and Western blotting. **K** PTC cells were transfected with sh-NC or sh-circTIAM1 or co-transfected with sh-circTIAM1 and miR-646 inhibitor. Protein levels of HNRNPA1 were detected by Western blotting. Data represent the mean ± SD. Three independent assays were performed (**D**–**F**, **H**, **I**) (*p < 0.05; Student’s *t*-test).

As previous studies have shown that circRNAs can act as microRNA sponges and ultimately influence the expression of downstream genes, the possible targets of miR-646 and circTIAM1 were investigated. RNA-seq performed with stable circTIAM1-knockdown TPC-1 and control cells (Fig. 5A) revealed 929 downregulated and 468 upregulated mRNAs (Fig. 5B). The Gene Ontology and Kyoto Encyclopedia of Genes and Genomes analyses results are shown in Fig. S3A–S3D. Bioinformatics analysis was then conducted to predict the potential miR-646 targets using Targetscan (binding site≥2) and Mirtarbase, and ten genes (HNRNPA1, MAP3K7, PIK3R1, CHAC1, TMEM245, CREBRF, PLAG1, RNF149, SLIT3, and ZNF704) were filtered by overlapping the potential miR-646 targets and downregulated genes identified by RNA-seq (Fig. 5C). Next, the expression levels of these ten genes were determined in sh-circTIAM1 or sh-NC TPC-1 and B-CPAP cells by RT-qPCR (Fig. 5D, E). HNRNPA1, TMEM245, and MAP3K7 genes were downregulated by circTIAM1 knockdown, and PTC cells transfected with si-HNRNPA1 exhibited significant apoptosis (Fig. 5F and Fig. S4A). As illustrated in Fig. 5G, HNRNPA1 contains 8-mer-1a and 7-mer-m8 potential sites for binding of miR-646. The dual-luciferase reporter assay indicated that miR-646 mimics markedly reduced the luciferase activity of the HNRNPA1-WT 3ʹ-UTR reporter compared with controls, whereas this phenomenon was not observed with the HNRNPA1-MUT 3-UTR reporter (Fig. 5H). Furthermore, HNRNPA1 expression was detected in PTC cells transfected with miR-646 mimics or inhibitor at the RNA and protein levels. HNRNPA1 was downregulated by miR-646 mimics and upregulated by miR-646 inhibitor, indicating that HNRNPA1 is a direct target of miR-646 (Fig. 5I, J). The expression of HNRNPA1 was downregulated by circTIAM1 inhibition and could be partially reversed by miR-646 inhibitor, as shown in Western blot (Fig. 5K).

HNRNPA1 has important cancer-promoting effects in many tumors, but its function in PTC is rarely reported. To evaluate the function of HNRNPA1 in vitro, si-HNRNPA1 was designed and transfected into PTC cells. Transfection efficiency was confirmed by qRT-PCR (Fig. S4B). Transwell migration and wound healing assays were then conducted to reveal the critical role of HNRNPA1 in promoting PTC cell migration (Fig. S4C and D). In addition, inhibition of the proliferation ability was observed in cells with lower levels of HNRNPA1 by colony formation and CCK-8 assays (Fig. S4E, F). Taken together, these lines of evidence indicate that miR-646 may influence PTC progression via HNRNPA1.

### Overexpression of HNRNPA1 reverses the antitumor effects of miR-646 on PTC cells

**Fig. 6:**
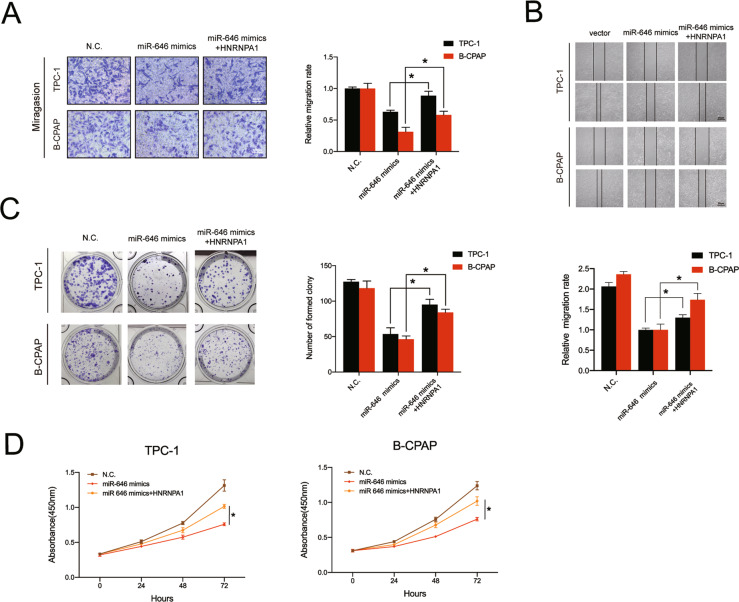
HNRNPA1 mediates the function of miR-646 in PTC cells. **A** The Transwell migration assay showed that the effect of miR-646 overexpression on cell migration was abrogated by high levels of HNRNPA1. Scale bars = 100 μm. **B** The migration capacity was measured by the wound healing assay with PTC cells. **C** the colony formation assay showed that HNRNPA1 overexpression abrogated the loss of proliferation ability of PTC cells with miR-646 mimics. **D** The decreased proliferative potential of cells with high levels of miR-646 was abrogated by HNRNPA1 overexpression, as demonstrated by the CCK8 assay. Data represent the mean ± SD. Three independent experiments were performed (**A**–**D**) (*p < 0.05; Student’s *t*-test).

To further investigate whether the antitumor effects of miR-646 are mediated by HNRNPA1 inhibition, TPC-1 and B-CPAP cells were co-transfected with HNRNPA1 overexpression plasmid and miR-646 mimics or NC. The results showed that overexpression of HNRNPA1 could markedly rescue the deficiency of migration ability in PTC cells transfected with miR-646 mimics (Fig. 6A, B). In addition, high levels of HNRNPA1 rescued colony formation and cell proliferation abilities, which were reduced by miR-646 mimics (Fig. 6C, D). Altogether, these findings reveal that miR-646 targets HNRNPA1 to prevent PTC progression.

### CircTIAM1 promote tumor growth in vivo

**Fig. 7:**
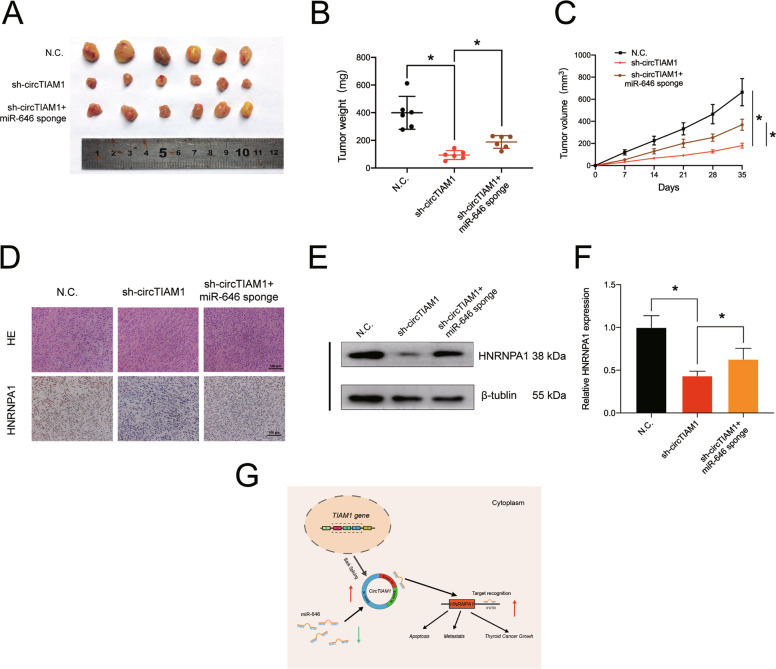
CircTIAM1 sponges miR-646 to promote tumorigenesis in vivo. **A** 1 × 10^7^ TPC-1 cells were injected into nude mice, and representative images of subcutaneous tumors were photographed after five weeks. **B** Tumor weights of mice were measured in each group after the mice were dissected (*n* = 6 per group). Data represent the mean ± SEM. *p < 0.05. **C** Tumor volumes were recorded every week after injection. Data represent the mean ± SEM (*n* = 6). *p < 0.05. **D** Immunohistochemistry assay was conducted to exhibit HNRNPA1 protein levels in tumors, and H&E staining was performed to show the tumor structure. Scale bars = 100 μm. **E** HNRNPA1 expression of tumors from different groups was detected at the protein level by Western blot assay. **F** HNRNPA1 expression of tumors was detected at the RNA level by qRT-PCR assay. **G** Schematic representation of the hypothesis of the circTIAM1/miR-646/HNRNPA1 axis.

As previous studies have revealed that circTIAM1 could promote tumorigenesis in PTC cell lines, we further examined its function in vivo. Human lentivirus-sh-circTIAM1 and miR-646 sponge were used to establish stable transfected TPC-1 cells with three groups (the control group, stable circTIAM1-inhibited group, as well as the circTIAM1 and miR-646 double-inhibited group). Cells were then injected subcutaneously into nude mice (6 mice per group) to establish a xenograft tumor model. The nude mice were well reared, and the subcutaneous tumor sizes were regularly recorded until the mice were sacrificed. As shown in Fig. 7A, B, the final average tumor sizes and wet weights were decreased with the transfection of sh-circTIAM1 but not with the transfection of NC, whereas higher proliferation capacity was observed in cells double-transfected with sh-circTIAM1 and miR-646 than the sh-circTIAM1-transfected group. In addition, the inhibition of circTIAM1 significantly affects the cell proliferation of PTC in vivo (Fig. 7C). Further, the relationship between circTIAM1 and HNRNPA1 was evaluated in vivo. The lack of circTIAM1 led to a decrease in the mean immunopositive area for HNRNPA1, which could be counteracted by inhibiting miR-646 (Fig. 7D). In addition, circTIAM1 knockdown reduced the expression of HNRNPA1 at protein and mRNA levels, and inhibition of miR-646 reversed this effect (Fig. 7E, F). In conclusion, these results indicate the tumor-promoting effects of elevated circTIAM1 in PTC in vivo via the modulation of miR-646 (Fig. 7G).

## Discussion

At present, surgery and iodine 131 radiotherapy are the main strategies for the clinical treatment of PTC, while for advanced and iodine-resistant PTC, there is still a lack of effective targeted drugs. Hence, investigating the mechanisms underlying PTC pathogenesis is important for the development of effective therapeutics. circRNAs are endogenous non-coding RNAs that may become important targets in anticancer therapy because of their widespread and tissue-specific expression and highly conserved features among mammals. Previous research has proven that certain circRNAs are associated with the development and progression of cancers, while their functions in PTC have been rarely studied.

CircTIAM1 is generated by the back splicing of exons 14–16 of the TIAM1 gene. It mainly comprises the protein-coding sequence of the TIAM1 mRNA. TIAM1 is a guanine nucleotide exchange factor that specifically regulates the Rac1 signaling pathway to mediate cell migration, adhesion, growth, and polarity. Evidence has confirmed that the TIAM1 gene plays an important role in a variety of tumor activities, such as colorectal cancer [20] and breast cancer metastasis [21]. In addition, high levels of TIAM1 have been reported to increase the risk of recurrence of thyroid cancer [22], and the knockdown of TIAM1 significantly inhibited the migration and invasion potential of PTC cells in vitro [23]. Thus, we hypothesized that circTIAM1 might be partly involved in tumor progression as a circRNA originating from TIAM1. In the present study, we showed that circTIAM1 was significantly upregulated in PTC tissues. Although circTIAM1 was not upregulated in the KTC-1 cell line, which may be due to the inherent biological and gene mutation differences between the various PTC cell lines, it was upregulated in the majority of PTC cell lines (K1, TPC-1, IHH-4, B-CPAP). In vitro gain- and loss-of-function experiments and in vivo xenograft experiments were conducted, further demonstrating the role of circTIAM1 in papillary thyroid cancer progression, which may provide an in-depth understanding of the pathology of PTC and provide novel perspectives for diagnosis and treatment.

With the continuous development of second-generation sequencing technology, accumulating research has focused on the mechanistic role of these circRNA-miRNA-mRNA competing endogenous RNA networks in many diseases, especially cancers [24–26]. In our study, we found that miR-646 has a high capacity to bind to circTIAM1. Furthermore, circTIAM1 can positively regulate the expression of HNRNPA1 (miR-646 target), leading us to propose a mechanism wherein circTIAM1 sponges miR-646 to contribute to PTC progression.

MiR-646 currently seems involved in cell proliferation and metastasis in a variety of tumors and was reported to mediate the VAMP2 and MDM2 genes at the post-transcriptional regulation level, thereby inhibiting the activity of ovarian cancer cells [27]. miR-646 could be reversely regulated by hypoxia-inducible factor-1α in pancreatic cancer to inhibit the expression of migration and invasion inhibitory protein, thus inhibiting tumor cell proliferation and invasion [28]. In addition, miR-646 targets FOXK1 to significantly inhibit cell proliferation and epithelial-mesenchymal transition in gastric cancer [29]. In this study, we reported for the first time that miR-646 is inversely related to HNRNPA1 expression, suppresses PTC proliferation and metastasis, and promotes tumor apoptosis.

HNRNPs are a family of RNA-binding nuclear proteins, of which HNRNPA1 is one of the most well-characterized members [30]. HNRNPA1 can regulate gene expression and cell signaling through its role in pre-mRNA and mRNA biogenesis [31]. As a pre-mRNA splicing inhibitor, HNRNPA1 regulates CD44 isoforms, and low levels of HNRNPA1 can significantly inhibit cell invasion and induce cell death in breast cancer [32]. Furthermore, HNRNPA1 knockdown significantly inhibited the overexpression of CD44v6 and caused an apparent decrease in the invasive capacity of tumor cells in hepatocellular carcinoma, while HNRNPA1 upregulation, caused by miR-490, promoted cell proliferation and migration [33]. In summary, there have been many studies showing that HNRNPA1 is closely related to a variety of malignant tumors, but so far, there has been little research on the correlation between HNRNPA1 expression and PTC progression. In this study, we verified HNRNPA1 as a tumor driver gene for PTC for the first time. By inhibiting the expression of HNRNPA1, we observed decreased migration and proliferation of PTC cells. Moreover, HNRNPA1 was shown to be a direct target of miR-646 and can reverse the antitumor effect of miR-646 in PTC cell lines.

In summary, we propose that targeting the circTIAM1/miR-646/HNRNPA1 axis is a potentially effective strategy for the treatment of PTC. In PTC tissues and cell lines, circTIAM1 showed an increased level and downregulated miR-646 by sponging HNRNPA1, leading to the promotion of PTC progression and metastasis. To the best of our knowledge, our work is the first to explore the role of circTIAM1 in PTC and may provide a novel perspective on the diagnosis and treatment of PTC.

## Methods

### Clinical specimens

In total, 60 pairs of PTC and paracarcinoma tissues were obtained from patients who undergo thyroid cancer surgery at Sir Run Run Shaw Hospital of Zhejiang University School of Medicine between January 2019 and January 2020. All patients have written informed consents before their specimens were used for scientific research. Tissues were collected and stored in liquid nitrogen immediately during the surgery. Patients receiving any radiotherapy before surgery were excluded. CircRNA microarray sequencing and bioinformatics analysis were performed using tissues from three typical PTC patients and the raw data has been uploaded to the GEO database (GSE168449).

### Cell culture and treatment

The human PTC cell lines TPC-1, B-CPAP, KTC-1, IHH-4, K1, and the human thyroid follicular epithelial cell line Nthy-ori 3-1 were kindly provided by Stem Cell Bank, Chinese Academy of Sciences or ATCC (Manassas, VA, USA) and confirmed by STR profiling analysis. No mycoplasma contamination was detected. HEK-293T cells were maintained in Dulbecco’s modified Eagle’s medium (DMEM) with 10% fetal bovine serum (Gibco, Grand Island, NY, USA). PTC and human thyroid follicular epithelial cell lines were cultured in RPMI-1640 medium supplemented with 10% fetal bovine serum (Gibco, Grand Island, NY, USA). B-CPAP cells cultured in RPMI-1640 medium, 10% fetal bovine serum additional and MEM Non-Essential Amino Acids Solution (NEAA 100X) (Invitrogen, 11140050). Cells were incubated under proper conditions at 37 °C with 5% CO_2_.

### Xenograft tumor models

Four-week-old female nude mice were subcutaneously injected with approximately 1 × 10^7^ stable TPC-1 cells (six mice per group). The lengths and width of tumor xenografts were measured weekly using Vernier calipers and the volume was calculated according to the formula: volume = 0.5* (length × width^2^). After five weeks, xenograft tumors were harvested from mice and the weight of each tumor was measured with high-precision balance.

### Cell transfection

Human lentivirus-sh-circTIAM1, circTIAM1-overexpressing lentiviral plasmids, lentivirus-miR-646 sponge, and HNRNPA1-overexpressing lentiviral plasmids were purchased from HanBio (Shanghai, China). MicroRNA mimics and inhibitors were purchased from GenePharma (Shanghai, China). SiRNAs were designed and purchased from RiboBio (Guangzhou, China), and Lipofectamine iMax (Invitrogen, CA, United States) was selected for transfection. RNA was extracted to verify the transfection efficiency. The sequences are listed in Tables S2–S3.

### RNA extraction and quantitative real-time RT-PCR

Total RNA was extracted from PTC cell lines and clinical tissues and isolated with TRIzol reagent (Invitrogen, Carlsbad, CA, United States) and microRNAs were extracted with a miRNA Purification Kit (CWBIO, Jiangsu, China). qRT-PCR was performed using the Roche LightCycler 480II PCR instrument (Basel, Switzerland). Hifair II 1st Strand cDNA Synthesis SuperMix (Yeasen Biotechnology, Shanghai, China) and Hieff qPCR SYBR Green Master Mix (Yeasen) and were used for the circRNA and mRNA analyses, while miRNA cDNA Synthesis Kit (CWBIO) and miRNA qPCR Assay Kit (CWBIO) were used for microRNA analyses. The primers were purchased from Tsingke Biological (Hangzhou, China) and the sequences of the primers are listed in Table S4. The results were normalized to GAPDH for circRNA and mRNA and the internal standard control was U6 for microRNA.

### RNase R treatment

2.5 μg total RNA was extracted from TPC-1 and B-CPAP cells and then incubated for 15 min at 37 °C with or without 10 U RNase R (Geneseed, Guangzhou, China) for RNase R treatment. The expression of circTIAM1 and TIAM1 was then detected by qRT-PCR and results were normalized to GAPDH.

### Nucleic acid electrophoresis

2% agarose gel electrophoresis with TAE buffer was performed to separate circTIAM1 and linear TIAM1 mRNA in the cDNA and gDNA samples. GADPH act as a control. After electrophoresis at 130 V for 45 min, the bands were visualized by ultraviolet radiation using Super DNA Marker (CWBIO, Beijing, China).

### Fluorescence in situ hybridization (FISH)

Cy3-labeled circTIAM1 probes and fam-labeled miR-646 probes were purchased from General Biol (Anhui, China). Cell nuclei were counterstained with DAPI. Fluorescent In Situ Hybridization Kit (RiboBio) was used to determine the signals of the probes. Images were acquired using a digital slice scanner Pannoramic MIDI (3D HISTECH, Hungary). The sequences of these probes are listed in Table S5.

### Prediction of miRNA targets of circTIAM1

MiRNA binding sites of circTIAM1 were predicted using the databases TargetScan (http://www.targetscan.org/), miRanda (http://www.microrna.org/) and RNAhybrid (https://bibiserv.cebitec.unibielefeld.de/rnahybrid/). The filtering restrictions were as followed: (i) Number of estimated binding sites ≥2 and (ii) Total score ≥150 and Total energy < −15 kcal/mol and (iii) Minimum free energy (MFE) ≤ −25 kcal/mol.

### Dual-luciferase reporter assay

Dual-luciferase reporter plasmids were designed with pSI-Check2 vector plasmid containing the and renilla luciferase (hRluc) and firefly luciferase (fLUC) reporter and obtained from Hanbio. HEK-293T cells were cotransfected with a plasmid containing wild-type or mutant 3′-UTR of circTIAM1 or HNRNPA1 and miR-646 mimics or mimic-NC. After 48 h, hRluc activity and fLUC activity were measured with a Dual-Luciferase Reporter Gene Assay Kit (Beyotime, Shanghai, China). The hRluc/fLUC ratio was calculated and used for comparison between groups.

### CCK-8 assay and Colony formation assay

PTC cells were processed according to the experimental requirements and seeded into 96-well plates at a density of 3 × 10^3^ per well. After treatment for 0, 24, 48, and 72 h, 10 µL CCK-8 (Beyotime, Beijing, China) were added to each well. Then, absorbance was measured using a spectrophotometer (Thermo Fisher Scientific, Vantaa, Finland) at 450 nm after incubation for 2 h. For colony formation assay, cells were cultured in 12-well plates for eight days and washed with PBS twice. Then, clones were fixed with 4% paraformaldehyde for 20 min, and 0.1% crystal violet stain was used to Show the status of cell clones. The numbers of colony formation were counted under a microscope (Zeiss, Primovert). The experiments were repeated three times independently.

### Wound-healing assay

Transfected PTC cells were seeded in six-well plates, and after 24 h, the cells were scratched with a 200 μl pipette tip slowly and carefully (time 0 h). Then, the scratched area was washed twice with PBS, and representative images were captured after 24 h. The relative migration ratio was measured as the decreasing distance across the induced injury area, normalized to the 0 h control.

### Transwell migration assays

Cell migration was assessed with a Transwell chamber (8 μm; Millipore, Billerica, MA, USA). Briefly, Serum-free 1640 medium (200 μl) containing 2 × 10^5^ cells were seeded in the upper chamber. 500 μL 1640 medium containing 10% FBS was added to the lower chamber. After incubation for 24 h, the cells were fixed and stained with 0.1% crystal violet. Migrated cells were quantified and counted in three random fields using an inverted light microscope (Zeiss, Primovert).

### Apoptosis analysis

Cell apoptosis was determined using an Annexin V-FITC/PI kit (BD Biosciences, San Diego, CA, USA). Briefly, cells were seeded in six-well plates and digested using trypsin without EDTA. Then, cells were washed with PBS twice softly and stained with Annexin V-FITC/PI according to the manufacturer’s instructions. The cells were analyzed after 15 min of incubation at room temperature using a BD FACSCanto II flow cytometer (BD Biosciences, San Jose, CA, USA) and FlowJo software 10.4 (FlowJo, Becton, Dickinson & Company, CA, USA).

### RNA immunoprecipitation

RIP assay was performed using the RNA Immunoprecipitation Kit (BersinBio, Guangzhou, China). B-CPAP cells were transfected with Ago2 overexpression plasmid or control, and 1 × 10^7^ cells were lysed (200 μl) for each group plus protease inhibitors cocktail and RNase inhibitors and incubated with control IgG or an anti-ago2 antibody (Abcam, Cambridge, UK)-coated beads with rotation overnight at 4 °C. The purified RNA was used to evaluate circTIAM1 level through qRT-PCR assay.

### Western blotting

Total protein was extracted from cells and tissues using RIPA lysis buffer containing protease inhibitor (Fudebio, Hangzhou, China). Protein lysates were separated by SDS-PAGE gels and subsequently electrophoretically transferred to polyvinylidene difluoride (PVDF) membranes. Then the membranes were blocked for 1 h with 5% nonfat skim milk in TBST and incubated with specific primary antibodies at 4 °C overnight. Then, the membranes were washed with TBST and incubated with a secondary antibody for 1 h (Fudebio, Hangzhou, China). Anti-beta tubulin were purchased from Abcam (Cambridge, UK) and anti-HNRNPA1 antibodies were purchased from ABclonal (Wuhan, China).

### Statistical analyses

Statistical analyses were carried out with GraphPad Prism 8.2.1 (GraphPad Software, La Jolla, CA, USA). Unpaired Student’s *t*-test was used unless otherwise noted. Data are expressed as means with SDs. *P* values <0.05 were regarded as statistically significant.

## Supplementary information

Supplementary figure legends

Additional file 1 Table S1-5

Additional file 2 Figure S1

Additional file 3 Figure S2

Additional file 4 Figure S3

Additional file 5 Figure S4

cddiscovery-author-contribution-form

## Data Availability

The datasets supporting the conclusions of this article are available from the corresponding author on reasonable request.
