# Supplementary material for: CircRNA circTIAM1 promotes papillary thyroid cancer progression through the miR-646/HNRNPA1 signaling pathway
Source: Cell Death Discov. 2022 Jan 12;8:21. doi: 10.1038/s41420-021-00798-1 (PMC8755710; doi:10.1038/s41420-021-00798-1)
Supplement: Supplementary file 1 — Supplementary figure legends [file 41420_2021_798_MOESM1_ESM.docx]

**SUPPLEMENTARY FIGURE LEGENDS**

**Additional file 1: Table S1.** CircTIAM1 expression and its relationship with PTC patients. **Table S2.** ShRNA or siRNA sequences used in this research. **Table S3.** MiRNA mimics, and inhibitors sequences used in this research. **Table S4.** Primers and RNA sequences used in this study. **Table S5.** FISH probes sequences of CircTIAM1 and miR-646 in this research.

**Additional file 2: Figure S1.** The detaills list of circular RNA microarray analysis and the expression abundance of circTIAM1. **A**. The volcano plot of circular RNA microarray analysis. **B.** The detailed list of the top 50 differentially expressed circRNAs.

**Additional file 3: Figure S2.** The apoptosis function and transfection efficiency of miR-646 in PTC cell lines. **A**. TPC-1 and B-CPAP cells were transfected with various microRNA mimics or mimics N.C. After 48 h, the apoptosis rate of PTC cells was evaluated with Annexin V-FITC/PI staining. **B**. The transfection efficiency of PTC cells treated with miR-646 mimics or inhibitor. Data represent the mean ± SD (*P < 0.05 by Student’s t-test).

**Additional file 4: Figure S3.** Bioinformatic analysis of transcriptome sequencing of PTC cells transfected with sh-circTIAM1. **A.** The comparison map of the differentially expressed genes and all genes at GO Level2. **B.** GO analysis of downregulated genes in circTIAM1-inhibited cells is shown. **C** & **D.** KEGG analysis of downregulated genes in circTIAM1-inhibited cells.

**Additional file 5: Figure S4.** Silencing of HNRNPA1 suppresses PTC progression.

**A.** TPC-1 and B-CPAP cells were transfected with si-HNRPNA1 (or si-TMEM245 or si-MAP3K7 or N.C.). After 48 h, the apoptosis rate of PTC cells was evaluated with Annexin V-FITC/PI staining. **B.** HNRNPA1 expression could be sharply decreased by si-HNRNPA1 in PTC cells. **C.** Representative images indicating that low level of HNRNPA1 inhibit cell migration by wound-healing assay. **D.** PTC cells were transfected with si-HNRNPA1 and showed worse migration ability. Scale bars=100 μm. **E-F.** The ability of clone formation and cell proliferation was decreased in PTC transfected with si-HNRNPA1. Data are from three independent experiments (mean ± SD) (**A-F**) (*P < 0.05 by Student’s t-test).
