# Supplementary material for: CircRNA circTIAM1 promotes papillary thyroid cancer progression through the miR-646/HNRNPA1 signaling pathway
Source: Cell Death Discov. 2022 Jan 12;8:21. doi: 10.1038/s41420-021-00798-1 (PMC8755710; doi:10.1038/s41420-021-00798-1)
Supplement: Supplementary file 2 — Additional file 1 Table S1-5 [file 41420_2021_798_MOESM2_ESM.docx]

**Table S1**

| **Table S1** Relationship between circTIAM1 expression and the clinical pathological characteristics of 60 PTC patients | | | | |
| --- | --- | --- | --- | --- |
| Characteristics |  | CircTIAM1 expression | | p Value^†^ |
|  | | Low | High |  |
| Gender | |  |  |  |
| Male | | 9 | 8 | 0.774 |
| Female | | 21 | 22 |  |
| Age (years) | |  |  |  |
| ＜45 | | 18 | 14 | 0.301 |
| ≥45 | | 12 | 16 |  |
| Tumor size (cm) | |  |  |  |
| ＜1 | | 21 | 12 | 0.020^*^ |
| ≥1 | | 9 | 18 |  |
| TNM Stage | |  |  |  |
| I/II | | 25 | 14 | 0.021^*^ |
| III/IV | | 5 | 16 |  |
| Lymph node metastasis | |  |  |  |
| Negtive | | 19 | 7 | 0.002^*^ |
| Positive | | 11 | 23 |  |
| Extra-thyroidal Extension | |  |  |  |
| Negtive | | 27 | 21 | 0.053 |
| Positive | | 3 | 9 |  |
| Nodular Goiter | |  |  |  |
| Negtive | | 21 | 15 | 0.114 |
| Positive | | 9 | 15 |  |
| ^†^Peason’schi-squared test | | | | |
| ^*^p<0.05 | |  |  |  |

**Table S2**

| **shRNA or siRNAs** |  |
| --- | --- |
| CircTIAM1 shRNA | GAGUCAGAUGCAGGGGAAA |
| HNRNPA1 si | GCCGTGGTGGTGGTGGATA |
| TMEM245 si | GGACAGAAGTTGCATGTCA |
| MAP3K7 si | GGAGTGGCTTATCTTCACA |

**Table S3**

| **Mimics and inhibitors** |  |
| --- | --- |
| miR-646 mimics | 5'-3' AAGCAGCUGCCUCUGAGGC  3'-5' CUCAGAGGCAGCUGCUUUU |
| NC mimics | UUCUCCGAACGUGUCACGUTT |
| miR-646 inhibitor | GCCUCAGAGGCAGCUGCUU |
| NC inhibitor | CAGUACUUUUGUGUAGUACAA |

**Table S4**

| **Primer for qPCR** |  |  |
| --- | --- | --- |
| MiR-646 | F | ATCAGGAGTCTGCCAGTGGA |
|  | R | Universal R Primer (CWBIO) |
| U6 | F | CTCGCTTCGGCAGCACA |
|  | R | AACGCTTCACGAATTTGCGT |
| TIAM1 mRNA | F | ACAGCTGCTGATACTTACGGG |
|  | R | GCGTCAGCAGCACGATTATT |
| CircTIAM1 | F | CAATAATCGTGCTGCTGACGC |
|  | R | CTTTGTACAGCTGGGTTGCTG |
| β-actin | F | AGAGCTACGAGCTGCCTGAC |
|  | R | AGCACTGTGTTGGCGTACAG |
| GADPH | F | AGGGCTGCTTTTAACTCTGGT |
|  | R | CCCCACTTGATTTTGGAGGGA |
| Human HNRNPA1 | F | GCTCACGGACTGTGTGGTAA |
|  | R | GGCCTTGCATTCATAGCTGC |
| Human TMEM245 | F | CTCCAGAGCGCATTCTTAGGT |
|  | R | CTCCAGAGCGCATTCTTAGGT |
| Human PIK3R1 | F | AAGAAGTTGAACGAGTGGTTGG |
|  | R | GCCCTGTTTACTGCTCTCCC |
| Human MAP3K7 | F | ATTGTAGAGCTTCGGCAGTTATC |
|  | R | CTGTAAACACCAACTCATTGCG |
| Human PLAG1 | F | AAACTTTTGAAAGCACGGGAGT |
|  | R | GGCGATCACAATGTTCGCAC |
| Human SLIT3 | F | GGCATCGTCGAAATACGCCTA |
|  | R | GCTGATGTCTATTCGCTTCAGTT |
| Human CHAC1 | F | TGAAGATCATGAGGGCTGCAC |
|  | R | GGTAACCAGGGTTCTGTGGG |
| Human RNF149 | F | GAGTGACTCTCGGCATGGAG |
|  | R | GAGTACCTGCCCCAGTTGTC |
| Human ZNF704 | F | TCAGAGGAACTAGACATGGACAA |
|  | R | CTTGAAGCTGTCAGCCGAGAG |
| Human CREBRF | F | ACCCACTTCAAGCACACAAAT |
|  | R | GGGTTGATCTTTACCTTTGCCT |

**Table S5**

| **Probes for FISH** |  |
| --- | --- |
| Cy3-circTIAM1 | CY3-TTTCTTTGTACAGCTGGGTTGCTGGTGA |
| Fam-miR-646 | FAM-GCCTCAGAGGCAGCTGCTT |
