# Supplementary figures and images for: CircRNA circTIAM1 promotes papillary thyroid cancer progression through the miR-646/HNRNPA1 signaling pathway
Source: Cell Death Discov. 2022 Jan 12;8:21. doi: 10.1038/s41420-021-00798-1 (PMC8755710; doi:10.1038/s41420-021-00798-1)

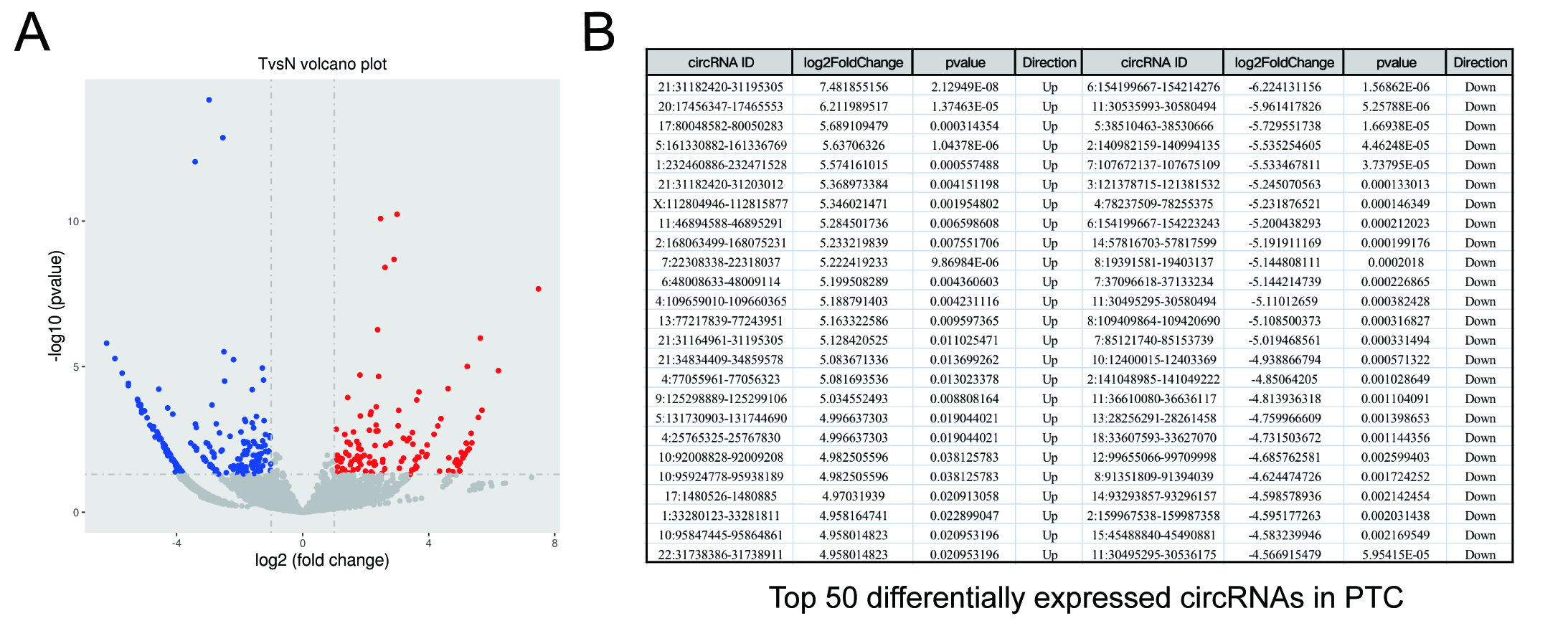

Supplement: Supplementary file 3 — Additional file 2 Figure S1 [file 41420_2021_798_MOESM3_ESM.tif]

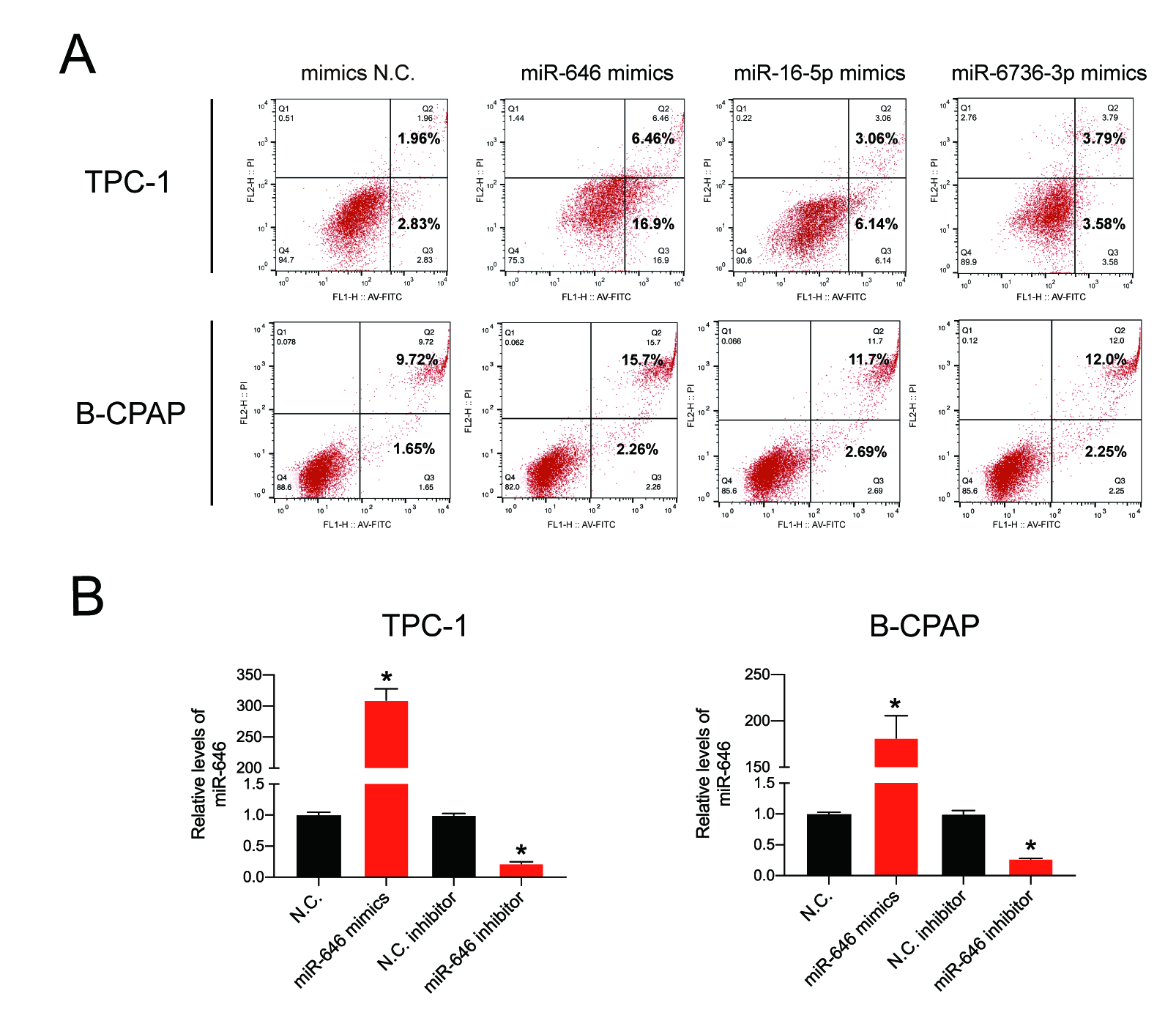

Supplement: Supplementary file 4 — Additional file 3 Figure S2 [file 41420_2021_798_MOESM4_ESM.tif]

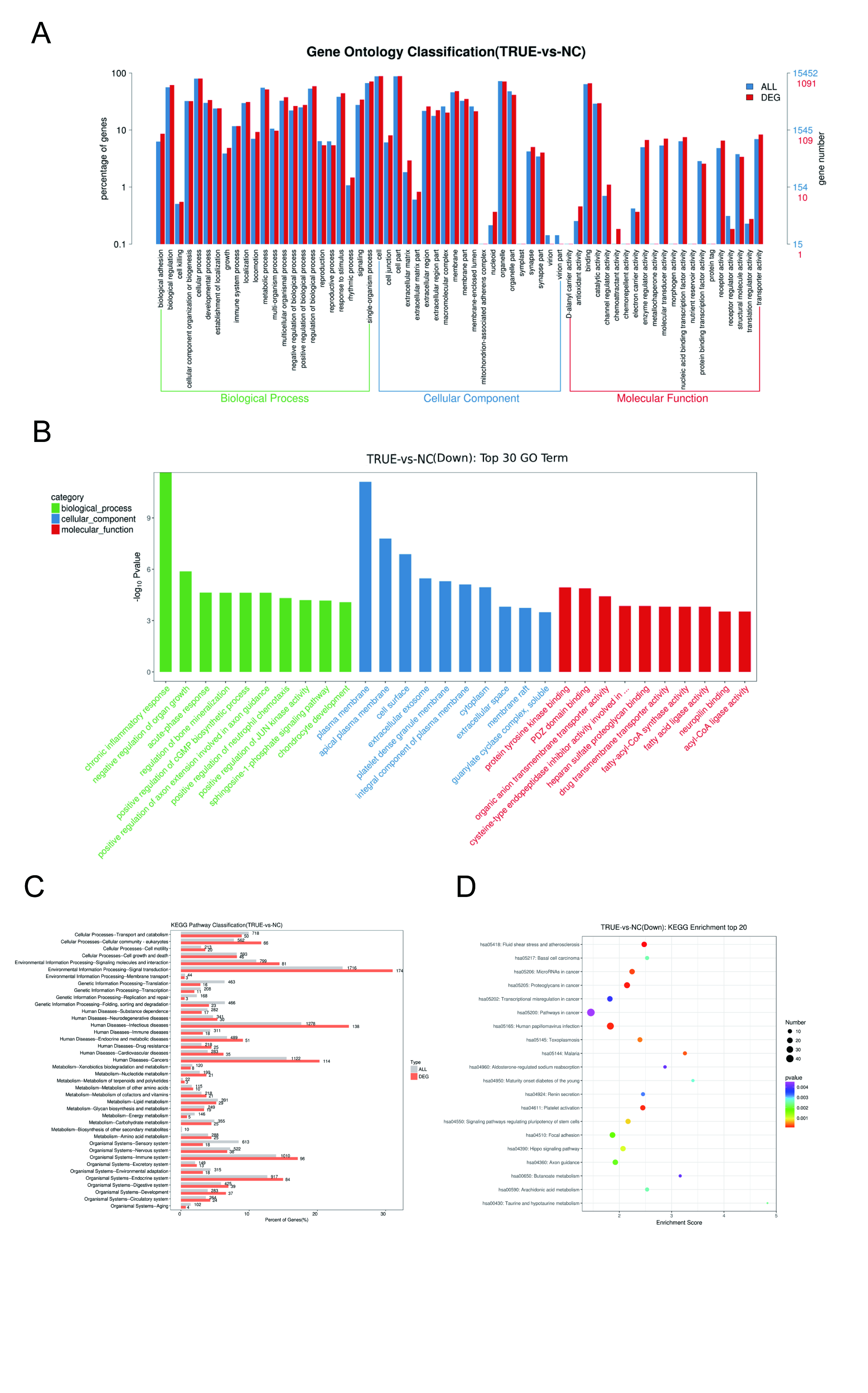

Supplement: Supplementary file 5 — Additional file 4 Figure S3 [file 41420_2021_798_MOESM5_ESM.tif]

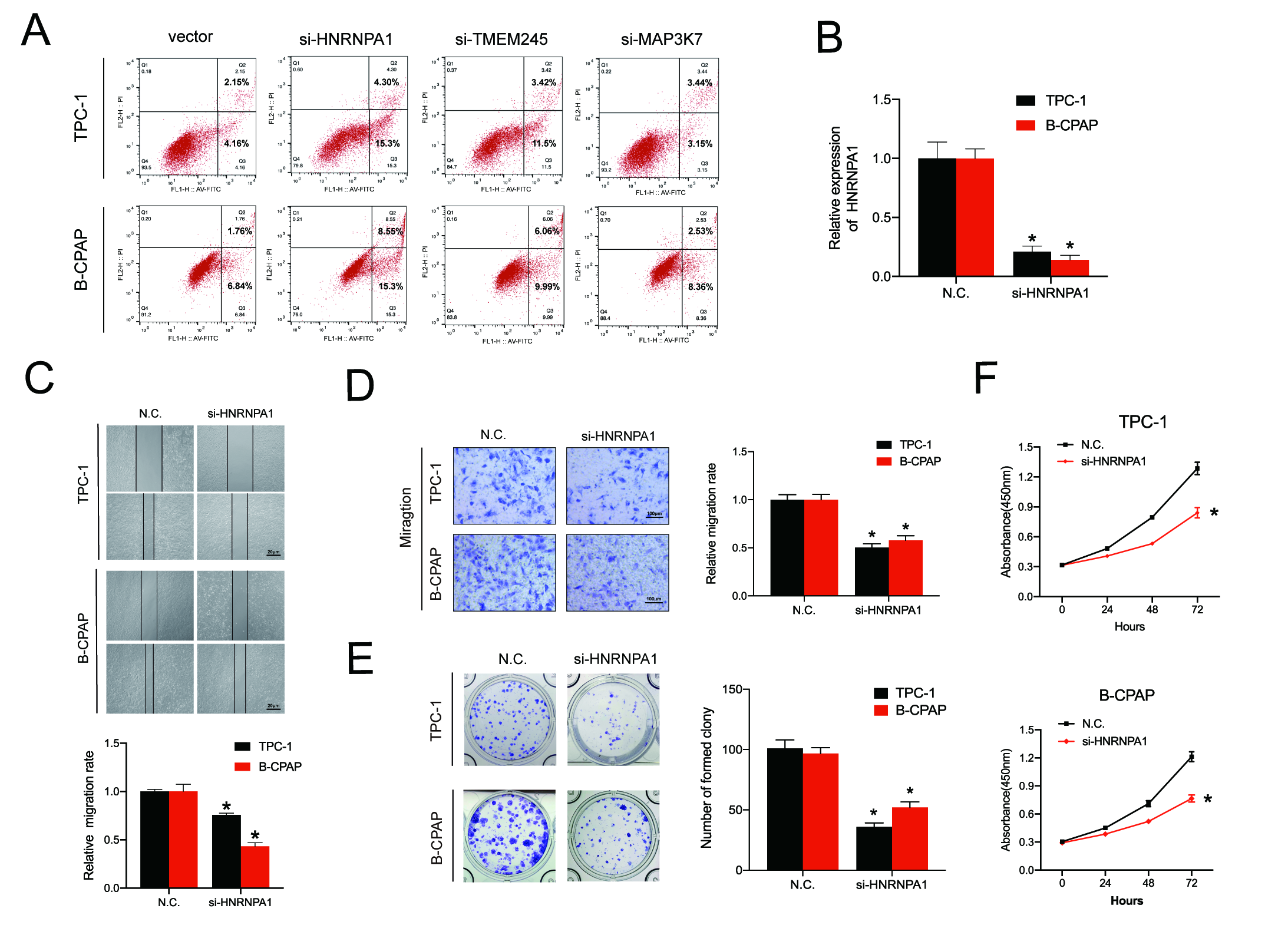

Supplement: Supplementary file 6 — Additional file 5 Figure S4 [file 41420_2021_798_MOESM6_ESM.tif]
